# Feasibility and cost of using mobile phones for capturing drug safety information in peri-urban settlement in Ghana: a prospective cohort study of patients with uncomplicated malaria

**DOI:** 10.1186/s12936-015-0932-8

**Published:** 2015-10-19

**Authors:** Vida Ami Kukula, Alexander A. N. Dodoo, Jonas Akpakli, Solomon A. Narh-Bana, Christine Clerk, Alexander Adjei, Elizabeth Awini, Simon Manye, Richard Afedi Nagai, Gabriel Odonkor, Christian Nikoi, Martin Adjuik, Patricia Akweongo, Rita Baiden, Bernhards Ogutu, Fred Binka, Margaret Gyapong

**Affiliations:** Dodowa Health Research Centre, Dodowa, Ghana; Centre for Tropical Clinical Pharmacology, College of Health Sciences, University of Ghana, Legon, Ghana; Department of Epidemiology and Disease Control, School of Public Health, University of Ghana, Legon, Ghana; INDEPTH-Network, Accra, Ghana; University of Science and Allied Sciences, Ho, Ghana

**Keywords:** Mobile telephone, Feasibility, Cost, Safety, Adverse events, Artemisinin combination therapy, Peri-urban and Ghana

## Abstract

**Background:**

The growing need to capture data on health and health events using faster and efficient means to enable prompt evidence-based decision-making is making the use of mobile phones for health an alternative means to capture anti-malarial drug safety data. This paper examined the feasibility and cost of using mobile phones vis-à-vis home visit to monitor adverse events (AEs) related to artemisinin-based combination therapy (ACT) for treatment of uncomplicated malaria in peri-urban Ghana.

**Methods:**

A prospective, observational, cohort study conducted on 4270 patients prescribed ACT in 21 health facilities. The patients were actively followed by telephone or home visit to document AEs associated with anti-malarial drugs. Call duration and travel distances of each visit were recorded. Pre-paid call cards and fuel for motorbike travels were used to determine cost of conducting both follow-ups. Ms-Excel 2010 and STATA 11.2 were used for analysis.

**Results:**

Of the 4270 patients recruited, 4124 (96.6 %) were successfully followed up and analyzed. Of these, 1126/4124 (27.3 %) were children under 5 years. Most 3790/4124 (91.9 %) follow-ups were done within 7 days of ACT intake. Overall, follow up by phone (2671/4124—64.8 %) was almost two times the number done by home visits (1453/4124—35.2 %). Duration of telephone calls ranged from 38 s to 53 min, costing between GH¢0.26 (0.20USD) and GH¢41.70 (27.USD). On the average, the calls lasted 3 min 51 s (SD = 3 min, 21 s) costing GH¢2.70 (0.77USD). Distance travelled for home visit ranged from 0.65 to 62 km costing GH¢0.29 (0.20USD) and GH¢279.00 (79.70USD). Thirty-two per cent (1128/4124) of patients reported AEs. In total, 1831 AE were reported, 1016/1831(55.5 %) by telephone and 815/1831 (44.5 %) by home visits. Events such as nausea, dizziness, diarrhoea, and vomiting were commonly reported.

**Conclusion:**

Majority of patients was successfully followed up by telephone and reported the most AEs. The cost of telephone interviewing was almost two times less than the cost of home visit. Telephone follow up should be considered for monitoring drug adverse events in low resource settings.

## Background

Information and communication technology has the propensity to improve a country’s health delivery system leading to positive implications for its health policy strategy [1]. An increasing number of countries in the world are using mobile communications to handle and address healthcare needs including behaviour change communication, training, education and awareness creation, data collection and monitoring in remote settings, disease and outbreak tracking, diagnosis and treatment [2]. The use of mobile phones to monitor health outcomes in low resourced settings, including Ghana will go a long way to reduce cost and maximize data collection within the health delivery system.

Malaria management in Africa is drastically hampered by poor, incomplete and untimely data on the incidence of disease and resource distribution and use, resulting in poor planning and implementation of interventions to ensure its control and elimination. To effectively deal with and sustain malaria control, Africa needs a quality health management system that can monitor malaria drug efficacy and safety. This requires timely and accurate data that is based on an effective and efficient communication network between health service providers and their clients [3]. Mobile health (mhealth) is the use of portable communication devices for creating, storing, retrieving and transmitting data between health service providers and their clients to improve the quality of care and patient safety [4, 5]. Decreasing cost and increasing network coverage has opened up more opportunities for many people in developing countries to access and use mobile phones and other communication gadgets. With the introduction of many and affordable wireless networks. Africa has indeed witnessed a significant improvement in communication in many of its rural communities and Ghana is no exception [6, 7]. Growing evidence suggests that mobile communication based health has the potential of radically improving healthcare in the most remote and less endowed communities in the world [8]. The use of mobile phone makes it easy for frontline health workers to use mobile technology rather than the traditional paper-based reporting system to gather and present health data with its inherent delays [9].

The growing need to capture data on health and health events using faster and efficient means to enable prompt evidence-based decision-making is making the use of mobile phones for health an alternative to traditional paper means of data capture [10, 11]. Growing mobile phone usage in Africa therefore offers the opportunity to explore its use in non-traditional modes of gathering data on health in resource-constrained settings, particularly on safety [12]. The use of medicines within the public and private health care system to treat large numbers of people presents the opportunity to generate real-life data on the safety and effectiveness of these medicines. Monitoring and documenting the safety of artemisinin-based combination therapy (ACT) is critical for public health programmes as any harm to a few patients, even if unrelated to the actual medicine administered, can impact negatively on the credibility, adherence to and success of any health programme [13, 14], as well as adherence to any recommended treatments.

Over the years, spontaneous reporting of adverse events has been the easiest way of monitoring drug safety. However, spontaneous reporting is associated with serious under-reporting and more active methodologies, including cohort event monitoring (CEM) are currently recommended by the World Health Organization (WHO) to complement spontaneous reporting [15–17]. CEM is a prospective, observational cohort study of adverse events associated with one or more medicines [18]. It is an active form of safety surveillance and records all clinical events and not just suspected adverse reactions. In developing countries, CEM provides the possibility of obtaining more complete safety data on medicines. Although more expensive and intensive than spontaneous reporting, the CEM method offers numerous advantages, including the ability to obtain denominator values, calculate incidence rates, demonstrate the absence of harm, and provide indications of the risk factors for adverse reactions [19]. CEM studies in Africa usually involve follow-up of patients through home visits, a costly and time-consuming approach fraught with logistical challenges. Mobile phones, on the other hand could provide a convenient, safe and cost-effective alternative for collecting safety data in real-life settings [20].

Mobile phones in Africa are evolving from simple communication tools into service delivery platforms, as seen with mobile money (M-Pesa) in Kenya. This has shifted the development paradigm surrounding mobile phones from one that simply reduces communication and coordination costs to one that could transform lives through innovative applications and services. Over 60 % of Africans in cities and small villages own mobile phones [20, 21]. Africans are buying mobile phones at a world record rate, with uptake soaring by 55 % in 5 years. Mobile subscriptions in Africa rose from 54 million to almost 350 million between 2003 and 2008, the fastest growth in the world. On average there are now 60 mobile subscriptions for every 100 people in the world. In developing countries overall, the figure stands at 48 for every 100 people. In Africa, penetration rates range from 16 % in Central African Republic, 43 % in Cameroon, to 84 % in South Africa [21]. A previous study showed that toll-free mobile phones offer a practical means of reporting adverse reactions to anti-malarial drugs and may be a model of choice in Africa [21, 22], where mobile telephone penetration and coverage is already driving innovation in agriculture and commerce. [22, 23] In a study by Fraser and Blaya, they concluded that the cost of mobile technology especially phones keep diminishing, making them the best alternatives for overcoming traditional obstacles to deliver health services to less resourced communities [24].

In Ghana, the mobile phone penetration rate at the end of August 2011 stood at 80.5 % [25]. The high penetration rates make the mobile phone a relevant and appropriate tool for improving health care delivery. According to the Dodowa Health and Demographic Surveillance System (HDSS) data, approximately 62 % of households in the surveillance area own mobile phones [26]. In recognition of the challenges of post-registration safety monitoring and limitations of spontaneous reporting, the INDEPTH Network Effectiveness and Safety Studies (INESS) on anti-malarial was initiated in Ghana, Tanzania, Mozambique and Burkina Faso with funding from the Bill and Melinda Gates Foundation. In Ghana, these studies were conducted in three HDSS sites, including Dodowa. The platform has strengthened efforts to document and report adverse events (AEs) in resource-limited settings using CEM to follow-up patients treated with ACT for uncomplicated malaria. This study sought to examine the feasibility and cost of using mobile phones vis-à-vis the traditional home visits to monitor AEs associated with anti-malarial for uncomplicated malaria in peri-urban settings in Ghana.

## Methods

### Study area

The Dangme West district, now Ningo-Prampram and Shai-Osudoku districts, is one of the peri-urban districts in the Greater Accra Region of Ghana. It covers a land surface area of 1529 square kilometres with mainly coastal savannah vegetation. The district is divided into four sub-districts and seven area councils for administrative purposes. There are 24 static health facilities and up to 20 outreach clinics delivering services in the district. Malaria is endemic in the district and is the leading cause of outpatient attendance. The Dodowa Health Research Centre (DHRC) maintains a HDSS, a longitudinal population registration system that serves the district. Vital demographic events such as pregnancies, births, deaths, and migration are updated bi-annually. Verbal autopsies are conducted to elucidate the circumstances surrounding and possible causes of all recorded deaths. In order to enhance spatial analysis of the data being collected, geographic information system (GIS) has been incorporated into the HDSS activities.

### Study design and sampling

This paper analysed CEM data gathered under real life conditions from February to December 2011. The CEM is the second aim of a multicentre phase IV INESS project that looked at a large cohort of about 10,000 participants from Ghana, Tanzania, Mozambique, and Burkina Faso. It was a prospective, cohort, non-interventional study intended to monitor the safety of the anti-malarial drugs using the spontaneous reporting system and cohort event monitoring under real life conditions. The data from the Dodowa HDSS, one of the research sites in Ghana that participated in the INESS project, were analysed. Patients prescribed anti-malarial were enrolled and followed up between day three (3) and day seven (7) of enrolment and all adverse events recorded. Patients were given the opportunity to report all adverse events of concern to the study team up to 28 days post drug administration. This process provides a real-life safety data in real time and complements the data obtained from spontaneous adverse event monitoring system.

Patients were recruited from both public and private health facilities in the district. All patients prescribed ACT for suspected or confirmed uncomplicated malaria were eligible for enrolment into the cohort. Patients were excluded if they had severe malaria, were in the first trimester of pregnancy or declined to offer consent. Pre-treatment questionnaires were administered to those who consented to participate at the time of recruitment at the health facility. Children 12–17 years assented and their parents/guardians gave consent. For those below 12 years, the parents/guardians consented for them to be included and the parents/guardians were interviewed on follow-up. To prevent loss of any data relating to ACT and also to ascertain the treatment of malaria in the districts, all patients presenting with uncomplicated malaria and prescribed an anti-malarial were recruited into the CEM study regardless of the actual anti-malarial prescribed. However, only patients prescribed an ACT were included in the analyses. Data collected included basic demographic information (age and sex), symptoms experienced in the preceding 5 days, drugs (including herbal preparations) taken within the preceding 14 days, dosage of drugs taken, indications for use of the drugs, and the start and end dates for taking the drugs. The prescribed ACT and any other drugs dispensed on the day of enrolment were recorded. The patient’s home address, contact phone number(s) and/or phone number of a close contact were also recorded. The phone numbers were validated by test calls before the patients left the health facility to ensure correctness and availability of the numbers. Patients who provided phone numbers and agreed to be followed up on phone were shown the prescribed ACT and informed that questions asked during the follow-up interview will be in relation to the prescribed ACT only. This was important and ensured that patients recognized that information to be collected during follow-up interviews was based on the ACT they had been prescribed.

### Follow-up

The routine procedure of the health facilities for monitoring adverse events is the use of the spontaneous adverse event monitoring system where patients are expected to come back to the facility to report any event experienced after drug intake. As part of the cohort event monitoring study protocol for monitoring and documenting these events, we use telephone calls and where this is not available, field officers went to the homes of the mothers or caretakers on motorbikes to conduct the interviews and document the events. Also, as part of the routine practice of the Research Centre, all field officers’ conduct monitoring using motorbikes for home visits and by phone calls.

### Procedure for type of follow-up

**Fig. 1:**
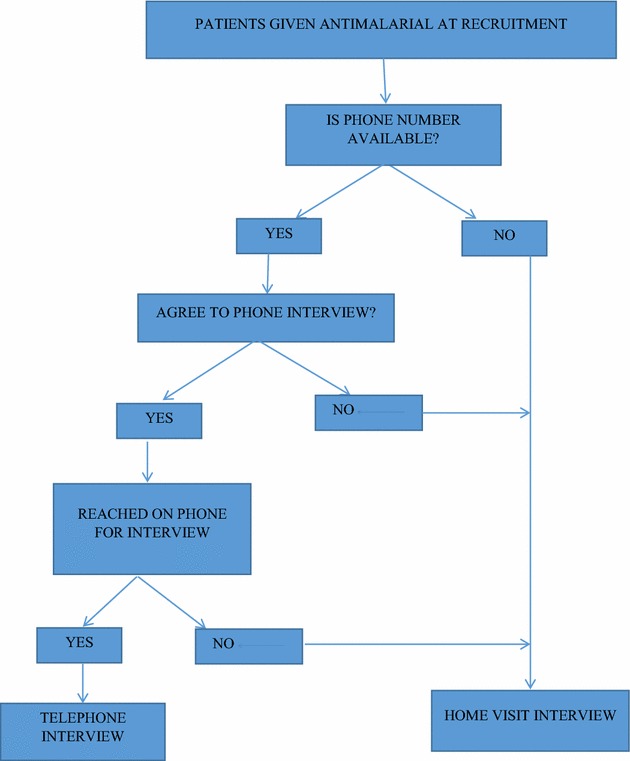
A *flow chart* showing how the patients recruited were followed-up via telephone or home visit

Two main types of follow-up procedures were used to document AEs experienced by patients prescribed ACT. Figure 1 shows the procedure by which patients were selected and followed-up using either telephone or home interview. Patients recruited for follow were asked to provide mobile phone numbers. Those without phone numbers were followed-up to their homes (as routinely done using motorbikes). Where the patients could not be reached on phone after three attempts on the interview date, they were followed-up to the homes and interviewed. Also those who had phone numbers but later disagreed to be interviewed on phone were also followed-up to the home.

Follow-up was carried out by field officers assigned to each of the seven area councils in the district. Field officers followed up patients recruited from health facilities located within their assigned area councils. Post-treatment questionnaires included questions to find out if patients ingested the ACT completely as prescribed. If the response was ‘yes’, the start and end dates for taking the ACT were recorded; if ‘no’, reasons for non-adherence were also recorded. For those who completed the treatment, new or worsening events and outcome for such events were recorded. A standard AE form was used to record the occurrence of all AEs regardless of severity and seriousness. The absence of any AE was also recorded to provide complete information on the safety of the medicines administered. All patients enrolled had only one active follow-up visit for safety assessment although patients had the team members’ phone details, permitting them to report AEs and obtain appropriate management for up to 28 days from the day of enrolment.

### Data processing and analysis

Completed questionnaires were returned to the field office weekly. These were checked by a research assistant and any inconsistencies identified were checked and corrected by contacting the field officer and/or patient. Data were double- entered using Epidata 3.1 and checked for inconsistency and coding errors. Data analysis was performed using MS-Excel 2010 and STATA 11.3 (Stata Corp, College Station, TX, USA). Data were presented using simple frequencies and percentages in graphs and tables. Descriptive statistics were used to present the types of follow-up done and the AEs reported. To determine the cost of using the telephone, pre-paid call credit was supplied to all the supervisors conducting the follow-up. Each field officer had a logbook to record each patient’s study number, telephone number, date of interview and duration of each call made. The per-second billing rate of 0.70 Cedis (USD0.20) per minute of the mobile network Scancom Company Network (MTN) was used irrespective of the mobile phone network that each field supervisor used. Although the district had six active mobile phone service networks, the mobile telephone network (MTN) was used because its coverage extended to the entire district. For assessing the cost of home visit, each field officer rode a motorbike and was given fuel per week and by kilometres covered. They logged in the distance travelled per visit and the standard Dodowa Health Research Centre cost of 0.45 Cedis (USD0.30) per kilometre for motorbike usage was used. Microsoft Excel 2010 was used to estimate the mean cost per interview conducted by phone and home visit.

### Implication and limitations

The authors of this paper are of the view that, this is the first time a study like this has been conducted in a low resource setting like Ghana. The results from this study are consistent with existing evidence that the mobile phone can be used as a tool for improving health outcomes in low resource settings. Notwithstanding this, the study presents some limitations. The authors did not use any rigorous systematic sampling procedure to select and assign patients to the type of follow-up. This may have introduced some discrepancies in the population selected and assignment of follow-up type. Also, we did not measure home distance variation among participants for home visit such as variations in the different routes leading to participants’ homes. For the phone calls, we did not use any standardized method of phone calling such as: call inquiry format, time of the day when calls were made and response of participants during busy periods.

## Results

### Background of participants

A total of 4270 patients prescribed anti-malarials from 21 health facilities in the Dangme West District (now Ningo-Prampram and Shai-Osudoku districts) were enrolled, 79/4270 (1.9 %) were lost to follow-up and 67/4270 (1.6 %) patients withdrew their consent. Analyses were performed on 4124 patients who were successfully followed up. Sixty-three per cent (2599/4124) of the patients were females and 1126/4124 (27.3 %) were children under 5 years of age. The median age of patients was 15 years with an inter quartile range of 29 years.

### Means of follow-up

Approximately 3586/4124 (86.9 %) of the patients provided mobile phone numbers at enrolment.

**Fig. 2:**
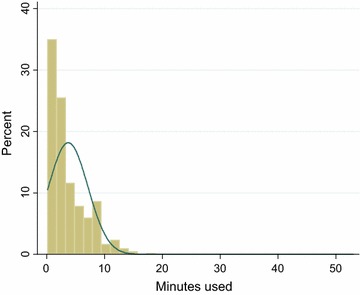
A graph showing the proportion of minutes used for follow-up by telephone call within 7 days of anti-malaria intake

Of those successfully followed up, 2671/4124 (64.8 %) were interviewed by telephone and the remaining 1453/4124 (35.2 %) were interviewed at home. On the whole, 3790/4124 (91.9 %) of the follow-up interviews were conducted within three to 7 days of ACT intake, of which 2532/3790 (66.8 %) was by telephone and 1258/3790 (33.2 %) was by home visits (Table 1). On average 34 interviews were done at follow-up per day. Of these, an average of 37 and 28 interviews were conducted by telephone and home visits per day, respectively. Eighty-two per cent (2189/2671) of these interviews were conducted at the first attempt to contact the patient and only 1 % (26/2671) of the patients were interviewed after three or more attempts (Fig. 2).

**Table 1:** Percentage of attempts at telephone call success within 7 days of anti-malaria intake

Days of follow-up	Phone n (%)	Home visit n (%)	Total n (%)
3–7	2532 (94.8)	1258 (86.6)	3790 (91.9)
8–28	139 (5.2)	195 (13.4)	334 (8.1)
Total	2671 (100.0)	1453 (100.0)	4124 (100.0)

One attempt at telephone call—82 %, two attempts at telephone call—14 %, three attempts at telephone call—3 %, and more than three attempts at telephone call—1 %

### Anti-malarial prescribed

Three-thousand and sixty of the 4124 (74.2 %) patients were prescribed artesunate-amodiaquine (ASAQ), 775/4124 (18.8 %) received artemether–lumefantrine (ALU) 208/4124 (5.1 %) received dihydroartemisinin–piperaquine (DHA/PQP) and 81/4124 (2.0 %) received other anti-malarial drugs (artesunate monotherapy, amodiaquine monotherapy or sulfadoxine–pyrimethamine). Of the 3060 patients who received ASAQ, 2098 (68.6 %) received the co-blister tablet formulation and 962 (31.4 %) received the fixed dose formulation.

### Adverse events (AEs)

Of the 4124 patients who were successfully followed up, 1128/4124 (27.3 %) reported one or more AEs. Among the 1128 patients who reported AEs, a total of 1831 AEs were recorded with the reporting rates being higher among telephone contacts than by home visit. 1007/1831 (55 %) of AEs were reported through telephone call and 824/1831 (45 %) during home visits. Patients prescribed ASAQ reported 1589/1831 (86.8 %) of AEs and 242/1831 (13.2 %) from those on ALU. The overall average number of AEs reported was 1.46 (SD = 0.77). Patients who received ASAQ reported an average of 1.49 (SD = 0.79) AEs and those who received ALU an average of 1.34 (SD = 0.68) was reported. The types and numbers of AEs reported for each of the ACT were comparable regardless of the type of follow-up used to collect the safety information. Common events such as nausea, dizziness, diarrhoea, and vomiting were reported by home visit and telephone call (Tables 2, 3).

**Table 2:** Top 20 reported adverse events among patients prescribed artesunate–amodiaquine during follow-up contact by phone and home visit

Adverse events^a^	Phone n, % (95 % CI)	Home n, % (95 % CI)	Total n, % (95 % CI)
Drowsiness	225, 26.2 (23.3–29.3)	227, 31.1 (27.8–34.6)	452, 28.4 (26.2–30.7)
Dizziness	100, 11.6 (9.6–14.0)	80, 11.0 (8.8–13.5)	180, 11.3 (9.8–13.0)
General weakness	93, 10.8 (8.8–13.1)	57, 7.8 (6.0–10.0)	150, 9.4 (8.0–11.0)
Vomiting	67, 7.8 (6.1–9.8)	86, 11.8 (9.5–14.3)	153, 9.6 (8.2–11.2)
Loss of appetite	41, 4.8 (3.4–6.4)	14, 1.9 (1.1–3.2)	55, 3.5 (2.6–4.5)
Stomach ache	38, 4.4 (3.1–6.0)	30, 4.1 (2.8–5.8)	68, 4.3 (3.3–5.4)
Fever	38, 4.4 (3.1–6.0)	20, 2.7 (1.7–4.2)	58, 3.7 (2.8–4.7)
Headache	29, 3.4 (2.3–4.8)	20, 2.7 (1.7–4.2)	49, 3.1 (2.3–4.1)
Cough	29, 3.4 (2.3–4.8)	15, 2.1 (1.1–3.4)	44, 2.8 (2.0–3.7)
Diarrhoea	23, 2.7 (1.7–4.0)	20, 2.7 (1.7–4.2)	43, 2.7 (2.0–3.6)
Nausea	19, 2.2 (1.3–3.4)	33, 4.5 (3.1–6.3)	52, 3.3 (2.5–4.3)
Palpitation	17, 2.0 (1.1–3.1)	22, 3.0 (1.9–4.5)	39, 2.5 (1.8–3.3)
Chills	12, 1.4 (0.7–2.4)	6, 0.8 (0.3–1.8)	18, 1.1 (0.7–1.8)
Restlessness	10, 1.2 (0.6–2.1)	7, 1.0 (0.3–2.0)	17, 1.1 (0.6–1.7)
Body pain	9, 1.0 (0.5–2.0)	7, 1.0 (0.3–2.0)	16, 1.0 (0.5–1.6)
Sleeplessness	9, 1.0 (0.5–2.0)	6, 0.8 (0.3–1.8)	15, 0.9 (0.5–1.6)
Body itchiness	8, 0.9 (0.4–1.8)	10, 1.4 (0.7–2.5)	18, 1.1 (0.6–1.8)
Sore mouth	7, 0.8 (0.3–1.7)	4, 0.5 (0.1–1.4)	11, 0.7 (0.3–1.2)
Cold	7, 0.8 (0.3–1.7)	2, 0.3 (0.1–1.4)	9, 0.6 (0.2–1.1)
Skin rashes	7, 0.8 (0.3–1.7)	1, 0.1 (0.0–0.7)	8, 0.5 (0.2–1.0)

^a^One patient could report more than one adverse event

**Table 3:** Top 20 reported adverse events among patients prescribed arthemeter–lumefantrine during follow-up contact by phone and home visit

Adverse events^a^	Phone N = 157 n, % (95 % CI)	Home N = 85 n, % (95 % CI)	Total N = 242 n, % (95 % CI)
Drowsiness	27, 17.2 (11.6–24.0)	30, 35.3 (25.2–46.4)	57, 23.6 (18.4–29.4)
Dizziness	16, 10.2 (6.0–16.0)	8, 9.4 (4.2–17.7)	24, 9.9 (6.5–14.4)
General weakness	14, 8.9 (5.0–14.5)	3, 3.5 (1.01–0.10)	17, 7.0 (4.1–11.0)
Vomiting	12, 7.6 (4.0–13.0)	8, 9.4 (4.2–17.7)	20, 8.3 (5.1–12.5)
Headache	9, 5.7 (3.0–10.6)	3, 3.5 (0.7–10.0)	12, 5.0 (3.0–9.0)
Body pains	9, 5.7 (3.0–10.6)	0, 0.0 (0.0–4.2)	9, 3.7 (2.0–7.0)
Nausea	7, 4.5 (2.0–9.0)	2, 2.4 (0.3–8.2)	9, 3.7 (2.0–7.0)
Fever	7, 4.5 (2.0–9.0)	1, 1.2 (0.0–6.4)	8, 3.3 (1.4–6.4)
Stomach ache	6, 3.8 (1.4–8.1)	0, 0.0 (0.0–4.2)	6, 2.5 (1.0–5.3)
Chills	5, 3.2 (1.0–7.2)	0, 0.0 (0.0–4.2)	5, 2.1 (1.1–4.8)
Diarrhoea	4, 2.5 (1.0–6.3)	1, 1.2 (0.0–6.4)	5, 2.1 (1.1–4.8,)
Sleeplessness	4, 2.5 (1.0–6.3)	1, 1.2 (0.0–6.4)	5, 2.1 (1.1–4.8)
Cough	3, 1.9 (0.4–5.4)	5, 5.9 (2.0–13.2)	8, 3.3 (1.4–6.4)
Body itchiness	3, 1.9 (0.4–5.4)	3, 3.5 (1.0–1.1)	6, 2.5 (1.0–5.3)
Palpitation	3, 1.9 (0.4–5.4)	3, 3.5 (0.7–10.0)	6, 2.5 (1.0–5.3)
Loss of appetite	3, 1.9 (0.4–5.4)	1, 1.2 (0.0–6.4)	4, 1.7 (0.1–4.2)
Body itchiness	3, 1.9 (0.4–5.4)	3, 3.5 (07–10.0)	3, 1.2 (0.3–4.0)
Cold	3, 1.9 (0.4–5.4)	0, 0.0 (0.0–4.2)	3, 1.2 (0.3–4.0)
Chest pain	2, 1.3 (0.1–5.0)	0, 0.0 (0.0–4.2)	2, 0.8 (0.1–3.0)
Loose stools	2, 1.3 (0.1–5.0)	0, 0.0 (0.0–4.2)	2, 0.8 (0.1–3.0)

^a^One patient could report more than one adverse event

### Costing of type of follow-up

**Fig. 3:**
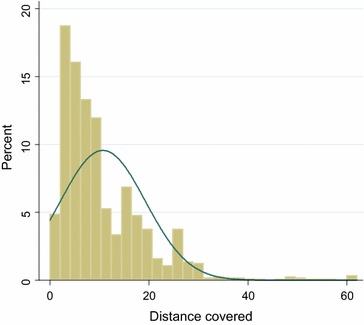
A graph showing the distance covered for home visits within 7 days of anti-malaria intake

For follow-up by telephone calls, the duration of calls ranged from 16 s to 53 min. These calls cost between 0.26 Cedis (USD0.20) and 41.70 Cedis (USD27.0), respectively. On average, the duration per call lasted 3 min 51 s (SD = 3 min 21 s) estimated at 2.70 Cedis (USD0.77). Most of the telephone calls lasted for 5 min (Fig. 2) at an estimated cost of 13.50 Cedis (USD3.86). For follow-up by home visit, the distance per visit ranged from 0.65 to 62 km. The distances travelled cost between 0.29 Cedis (USD0.20) to 279.00 Cedis (USD79.70). On average, the mean distance covered per visit was 10.7 (SD = 8.61) costing 51.6 Cedis (USD14.74). Most of the distances covered were within 10 km (Fig. 3). Using the cost per type of follow-up for the 1128 individuals who reported AEs, the total cost of AEs reported by telephone interviews (1016 AEs) was 2743.20 Cedis (USD783.77) and home visit (815 AEs) was 3926.3 Cedis (USD1122.37). The cost of telephone call interviews was almost two times less than that of the cost of home visits.

## Discussion

The objective of this paper was to examine the feasibility and cost of using mobile telephone vis-à-vis home visit to monitor adverse events (AEs) related to ACT for treatment of uncomplicated malaria in peri-urban Ghana. Considering that 4124 out of 4270 patients were successfully followed upon telephone with majority responding on first attempt at call in the largely rural Dangme West District (now Ningo-Prampram and Shai-Osudoku), this was quite remarkable. Approximately 3586/4124 (86.9 %) of the patients provided mobile phone numbers at enrolment This large number of patients in such a less endowed community like the Dangme West District confirms other studies that Africa has indeed witnessed a significant improvement in communication in many of its rural communities and Ghana is no exception [6]. Again, the provision of mobile phone numbers by such a large number on enrolment indicates that many of the patients had access and were connected to mobile network; thus supporting the growing evidence that mobile communication has the potential of radically improving healthcare in the most remote and less endowed communities in the world [7].

Reporting rates of AEs proved slightly higher among telephone contacts than by home visit. This shows that mobile phones are a feasible option for collecting safety data, more especially as it was possible for majority of the patients to be interviewed within 7 days of medicine intake. This result confirms findings of previous studies where follow-up using mobile phones proved feasible for collecting data and monitoring desired health outcomes [27, 28].

On the basis of comparative cost analysis, the study revealed that for the follow up by telephone, the duration of calls ranged from 16 s to 53 min and cost between 0.26 Cedis (USD0.20) and 41.70 Cedis (USD27.0) respectively while for the follow-up by home visit, the distance per visit ranged from 0.65 to 62 kilometres and cost between 0.29 Cedis (USD0.20) to 279.00 Cedis (USD79.70). The cost per follow-up through home visit was much higher compared with that of the use of mobile phone. The average cost of obtaining AE feedback from patients who received anti-malarial through the mobile phone platform was far cheaper (USD2.00 per phone call) than through home visits (USD3.20 per visit). Telephone call cost was almost two times less than the cost of home visit. Aside the cost, time is also of essence; it took less time to get the needed feedback for prompt and other necessary action when using the mobile phone than the home visit This confirms a previous study that assessed the effectiveness, safety and cost of diabetes tele-management system, and found telephone call use to be safer and more cost-effective than the traditional health care of frequent physical visits for every drug [29]. The findings of this study is at variance with the study by Ryan et al., who concluded that telephone monitoring expenses was an additional cost in the mobile group compared with paper-based monitoring [30, 31]. It is worth noting that the average cost for home visits estimated in this study covers only the direct cost of motorbike usage. Indirect costs such as travelling time, associated risk of riding the bike and the effects of the weather, were not included in the cost. While wear and tear on the phones may be apparent over time that of the motorbike cannot be overlooked. One advantage of this study confirms the findings of Asiimwe et al. that the use of mobile phone makes it easy for frontline health workers to use mobile technology rather than the traditional home visit and paper-based reporting systems to gather and present health data with its inherent delays [7].

Other advantages related to the telephone interviews are that they are brief, specific and convenient for patients who may feel uncomfortable reporting in face-to-face interviews. This confirms a previous study that examined whether an allied health professional telephone visit could safely substitute for an in-person clinic visit and concluded that patients accepted telehealth as the exclusive means of follow-up and nearly all patients expressed great satisfaction with the telephone follow-up method [32, 33].

The types, frequencies, order and proportions of events reported by both methods of follow-up elicited similar AEs reporting for each ACT dispensed. Important symptoms such as rashes, dizziness and sore mouth were picked by telephone call as well as for home visits for participants prescribed ASAQ. The top four AEs reported on telephone and at home visit for both ALU and ASAQ were similar. This corroborates the submission by Abedeji and colleagues that availability of a toll-free telephone line may facilitate pharmacovigilance and follow-up of response to medicines in a poor resource setting [19]. This is the first report from Ghana that provides evidence of cost of safety monitoring of AEs using telephone and confirms a study by Dodoo et al. who recommended that future research around methods of collection of AE data is transferable to rural settings [34].

## Conclusion

Majority of patients were successfully followed up by telephone and most AEs were on phone. The cost of telephone interview was almost two times less than the cost of home visit. Mobile phones thus represent an efficient, less costly and effective means of monitoring drug safety in resource-limited settings as demonstrated by this study in Ghana. Typical setbacks of spontaneous reporting, mainly under-reporting of AEs, is worse in low-resource settings such as Ghana and with the high mobile phone penetration, its use is the recommended method for collecting AEs as and when they occur, even in rural settings as propagated by Dodoo et al. [34].

Telephone calls and text messages may also reduce the problem of loss to follow-up which is unavoidable in the context of patients’ home visits [31]. Collecting AEs using the telephone can provide comprehensive real-life safety information on anti-malarials in resource-limited settings. The use of mobile phones to monitor health outcomes in Ghana and other developing countries in Africa will go a long way to reduce cost and maximize data collection within the health delivery systems of less resourced communities in the continent.
